# Topology of Plant - Flower-Visitor Networks in a Tropical Mountain Forest: Insights on the Role of Altitudinal and Temporal Variation

**DOI:** 10.1371/journal.pone.0141804

**Published:** 2015-10-29

**Authors:** Sandra Cuartas-Hernández, Rodrigo Medel

**Affiliations:** 1 Instituto de Biología, Universidad de Antioquia, Medellín, Antioquia, Colombia; 2 Departamento de Ciencias Ecológicas, Universidad de Chile, Santiago, Región Metropolitana, Chile; Universidade de São Paulo, Faculdade de Filosofia Ciências e Letras de Ribeirão Preto, BRAZIL

## Abstract

Understanding the factors determining the spatial and temporal variation of ecological networks is fundamental to the knowledge of their dynamics and functioning. In this study, we evaluate the effect of elevation and time on the structure of plant-flower-visitor networks in a Colombian mountain forest. We examine the level of generalization of plant and animal species and the identity of interactions in 44 bipartite matrices obtained from eight altitudinal levels, from 2200 to 2900 m during eight consecutive months. The contribution of altitude and time to the overall variation in the number of plant (*P*) and pollinator (*A*) species, network size (*M*), number of interactions (*I*), connectance (*C*), and nestedness was evaluated. In general, networks were small, showed high connectance values and non-nested patterns of organization. Variation in *P*, *M*, *I* and *C* was better accounted by time than elevation, seemingly related to temporal variation in precipitation. Most plant and insect species were specialists and the identity of links showed a high turnover over months and at every 100 m elevation. The partition of the whole system into smaller network units allowed us to detect small-scale patterns of interaction that contrasted with patterns commonly described in cumulative networks. The specialized but erratic pattern of network organization observed in this tropical mountain suggests that high connectance coupled with opportunistic attachment may confer robustness to plant-flower-visitor networks occurring at small spatial and temporal units.

## Introduction

Understanding the factors that determine the structure and dynamics of pollination networks is one of the unsolved questions in network ecology. Description of ecological networks as bipartite sets of interacting species allows the quantification of diverse descriptors of community complexity, which can reveal important properties of ecosystem structure and function [1,2,3,4]. Examination of tropical, temperate and arctic systems has shown consistent broad patterns of network structure [1,5]. However, the high environmental heterogeneity and sensitivity of species to small-scale variation in climatic conditions [6,7] often makes it difficult to predict the fine-scale patterns of occurrence and persistence of plant-animal interactions based on broad descriptors. In this regard, recent studies in plant—pollinator networks have examined the extent to which network structure varies across time and space. Results have revealed mixed patterns. For example, while some studies have shown slight variation in connectance estimates [8,9,10,11,12] and high and consistent nestedness values through time [8,9], other studies carried out at detailed temporal scales have found high and very variable connectances[13,14]. Complex temporal dynamics concerning the number and identity of species involved in pollination relationships has also been observed [8,9,13,14], and related to their phenology and abundance among other factors [10,15,16]. Furthermore, this complexity can occur even within a season, which suggests that to understand the effects of environmental variation on the structure of mutualistic networks, studies with appropriate temporal and spatial community resolution are needed [17]. To date, almost all ecological network studies are snapshots of limited temporal extent (i.e., a few years) that lack any finer temporal resolution [18]. At the same time, the influence of space, elevation, or precipitation gradients on network structure is by far less known (but see [17,19,20]), and studies analyzing simultaneously the temporal and spatial variation of network structure at small scale are almost nonexistent in the literature (but see [21]).

Studies assessing the temporal dynamics of plant—pollinator networks have been conducted mostly on temperate and arctic regions [8,9,10,11,13,14]. In these ecosystems, flowering plants have a well-defined flowering season [11] that contrasts with the lack of seasonality shown by tropical ecosystems. Even though plants and pollinators can in principle be active all year long, most species inhabiting tropical ecosystems have very specific habitat requirements and asynchronous phenologies that prevent their simultaneous occurrence in space or time(i.e., the ‘forbidden links’) [22]. In such cases, spatial and temporal data aggregation from cumulative samples (i.e., cumulative networks) may not necessarily represent adequately the real set of interactions and more realistic samplings should rely on records that capture species with truly coincident phenologies [13,14,17,23].

Consequently, the lack of data at finer temporal and spatial resolutions hinders the possibility to assess the role of the ecological factors underlying network structure and its variation in space and time [18].

In this work, we used a community-level approach to examine the influence of space and time on a tropical plant-flower-visitor network within a non-disturbed and continuous Andean mountain forest in Colombia. This mountain forest is characterized by having pronounced slopes and a bimodal precipitation regime; both factors that condition the observed set of interactions among species [6,7,24]. Additionally, a conspicuous change in the thermal band from mountain to alti-mountain at 2700 masl has been described for tropical Andean forests, with subsequent changes in the composition and structure of tree vegetation and microclimate, specifically in mean and minimum annual temperature [25].

Even though plants and animals can be active throughout the year in mountain forests, a strong altitudinal and temporal segregation in the distribution of species has been frequently observed, leading to a marked pattern of species turnover in space and time [25,26,27,28,29], including those involved in plant—pollinator networks (e.g., [9,14]). Thus, in general, we can expect that variation in environmental conditions associated with altitude and time create a highly heterogeneous mosaic of interactions due to changes in the composition of the interacting guilds. Specifically, in this study we asked the following questions: (1) Does network topology, measured as numbers of species, numbers of links, connectance, and nestedness vary through time or with elevation?; (2) Does network topology differ above and below 2700 masl where the thermal band changes?; (3) How consistent is the level of ecological specialization across altitude and time?; (4) Does the composition of plant and flower-visitor assemblages and therefore the identity of species interactions vary through time or along the altitude gradient?; and (5) Does network topology differ between individual vs. cumulative networks? To answer these questions, we analyzed a series of network metrics on eight plant- flower-visitor communities encompassing an elevation gradient of 800 m, during eight consecutive months in a Columbian mountain forest. We examined the pattern of change of network metrics across space and time, and assessed whether elevation or time is the main source of variation for network metrics in the overall system.

## Materials and Methods

### Study site

This study was carried out in the Reserva Natural La Mesenia, Paramillo, located at Jardín Antioquia, Colombia (5° 30' 11"N, 75° 51'7"E). The 1723 ha reserve is administrated by The Hummingbird Conservancy and comprises areas of conservation and areas for cattle grazing and agriculture. The conservation area is found mainly on pluvial cloud forest at steep zones from 2150 to 3100 masl (Cuesta *et al*. 2009). Precipitation exhibits a bimodal pattern with two rainy periods and two dry periods in the year [30].

### Sampling procedure

We traced 100 m transects perpendicular to the slope of the mountain at 100 m altitude intervals from 2200 to 2900 masl (T0 to T7), resulting in a total of eight 100 m transects. The presence of flowering plants and flower-visitors were recorded along transects on a monthly basis from October 2010 to May 2011, that includes four rainy months (mean monthly precipitation > 280 mm: October, November, April and May) and four dry months (mean monthly precipitation < 200 mm: December, January, February and March). This period included two rainy seasons and one dry season which allowed us to evaluate the effect of precipitation on the network structure. In total, we recorded data for 64 sampling units (8 transects x 8 months). At each visit, all flowering plant and flower-visitor species were recorded. An insect was considered as a flower-visitor when it was found on or inside the flower, regardless of their efficiency as a pollen vector. Sampling was performed through regular walks along each transect from 09h00 to 17h00 (8 hours of observation/day) paying special attention to interactions in the understory (~ 2 m height). The whole system was observed for a total of 512 h. Before the observation period, all plant species present along our transects were collected and identified at the Herbario Universidad de Antioquia (HUA). All insects visiting flowers were collected for identification at the laboratory because most of them were minute and their identification at a glance was unfeasible. Insects were morphotyped and in most cases, their identification was possible only to family or genus level, often with the assistance of specialists. Vouchers were deposited at the Entomological Collection of the Universidad de Antioquia (CEUA).

### Network analyses

Records of plant—flower-visitor interactions were used to construct 64 qualitative interaction matrices. The matrix elements *m*_*ij*_ are ones or zeros. Cells containing ‘‘1” represent an observed interaction between flower-visitor species *i* and plant species *j*, while cells with ‘‘0” represent absence of interaction [1]. We calculated a set of descriptive parameters from each matrix: number of plant species (*P*), number of insect species (*A*), total number of species (*S*), total number of interactions between plants and insects (*I*), network size (*M* = A*P), and a measure of network symmetry (*A*:*P*). Connectance (*C*) is the number of observed links divided by the total number of potential links between all species of plants and animals, *C* = (*I*/*M*) [4]. Specialization or generalization of interactions is characterized as the number of partners (or links). A plant or flower-visitor species interacting with only one partner was classified as specialist [31,32]. For each network we estimated the overall specialization/generalization level as the mean number of links per species (*L*) and the mean number of interactions per plant and animal species (*L*_P_ = *I*/*P* and *L*_A_ = *I/A*, respectively) [1]. Additionally, we estimated the percentage of specialist species for each network. Nestednesss was measured through the NODF metric based in the decreasing fill (DF) and paired overlap (PO) properties of a matrix. The absolute values of this metric are invariant to matrix size and shape, which is advantageous to estimate nestedness in small matrices [33,34], as in our case. We used the ANINHADO version Bangu 3.0.3 software [35]. NODF varies from zero for structures that have no degree of nestedness to 100 for perfect nestedness. A nested network implies that only a fraction of specialists interact with specialists, while the rest is expected to interact with moderate to extreme generalists, producing a highly connected core within the matrix [5].

To examine whether an observed network property differs from random, we constructed a null model based on an algorithm that randomized the total number of individual interactions observed in the original interaction matrix keeping constant the connectance [36]. All comparisons between observed and random values were performed using *t*-tests. The compared network indices were: (1) weighted connectance calculated by dividing linkage density by numbers of species in the network [37]; (2) weighted NODF [33,34]; (3) interaction strength asymmetry (ISA) which quantifies the average effect of each species on all its partners and is also ameasure of specialisation across both trophic levels. Positive values indicate higher dependence in the higher trophic levels [31]; and (4) Shannon’s diversity of interactions (H2), a network-level measure of specialization. It ranges between zero (no specialisation) and one (complete specialization).

We analyzed matrices consisting at least of two plant species and two flower-visitor species (i.e., *M* = 4) using the Bipartite Package v 1.17 [38] called by R Software v 2.12.2 [39] to obtain the parameters described above.

The contribution of altitude and time to the overall variation of every network descriptor was examined using an ANOVA. When variables did not did not fulfill the requirements of ANOVA (i.e., normality and homocedasticity) a Kruskal-Wallis test was used [40]. We tested for a linear or polynomial relationship of the network structure metrics with altitude or precipitation. Data on mean monthly precipitation was obtained from WorldClim Software v 1.4 [41]. This software provides a set of global climate grids with a spatial resolution of about one square kilometer. To investigate whether network topology differed between low and high altitude transects, (2200–2700 masl versus 2800–2900 masl) we performed *t*-tests on parameters. When the within group variances of a network property were not identical for low and high elevation groups, we used Welch’s approximate *t*-statistic to assess the significance of the difference, appropriate when samples have unequal variances [40].

To assess the similarity of species composition between networks, we performed SIMPER (Similarity Percentage) analysis [42]. This analysis provides a Bray-Curtis dissimilarity measure (multiplied by 100) and detects common species (i.e., core species) and rare species occurring exclusively at specific altitudes or months. These analyses were performed using PAST Software (Paleonthological Statistic software v. 2.12) [43]. Finally, we pooled data of all species and interactions recorded during the eight months on a per elevation basis (“altitude” networks), and pooled species and interactions occurring at all altitudes on a per month basis (“time” networks). We calculated the same set of descriptive parameters in cumulative networks as in individual matrices and tested for differences using a Kruskal-Wallis test [40].

## Results

### Descriptive network properties

**Fig 1:**
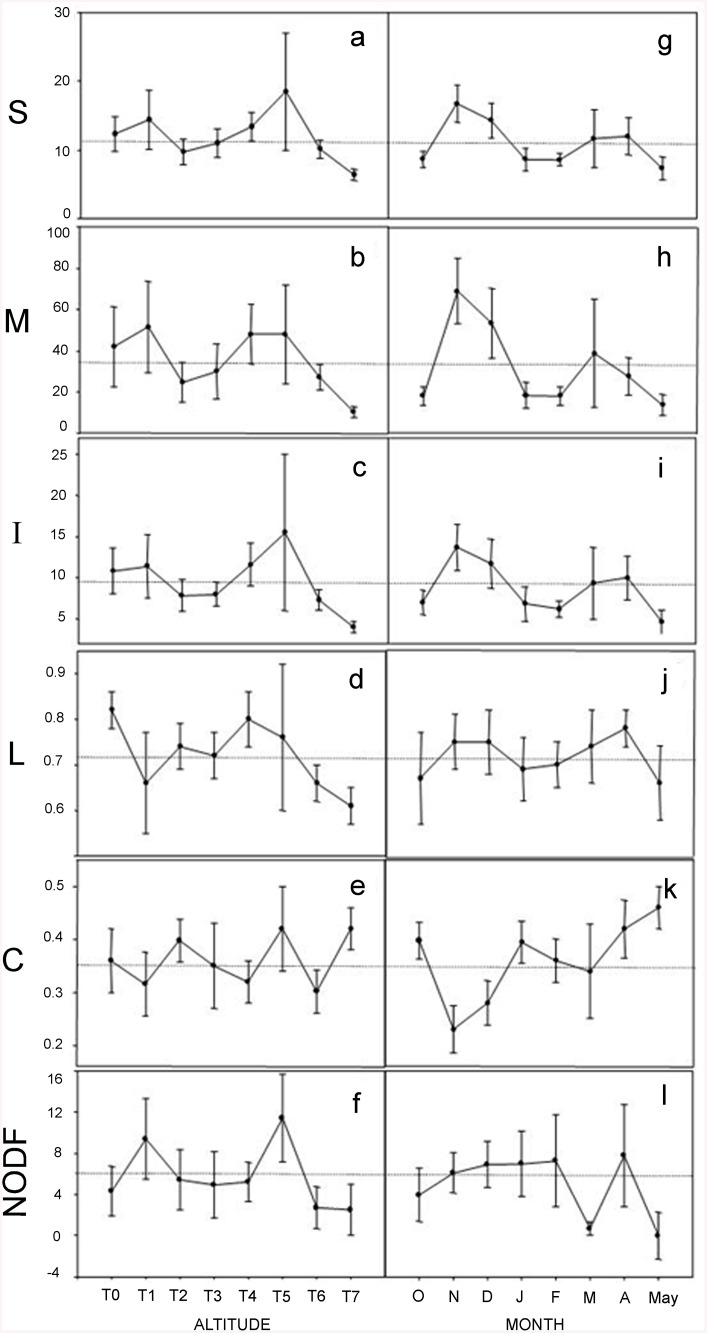
Variation in topological properties estimated from 44 plant-flower visitor networks in a cloud forest in Antioquia, Colombia. Means ± standard errors are shown for networks at each altitude (boxes A-F) and month (boxes G-L). (A, G) total number of species (*S*); (B, H) network size (*M*); (C, I) total number of interactions between plants and insects (*I*); (D, J) mean number of links per species (*L*); (E, K) Connectance (*C*); (F, L) Nestedness (Metric NODF). Horizontal lines inside boxes indicate the mean value of variables for the 44 networks.

We retrieved 44 out of 64 potential networks during the eight months of this study; 20 cells had no flowering plants or flower-visitor species. In total, 42 flowering plant species and 75 flower-visitor species were recorded: the list of plant and pollinator species in the overall system is reported in [44]. Metrics for the 44 analyzed networks are listed in S1 Table. The number of networks on the altitude samples ranged from three to seven and the number of networks on monthly samples ranged from two to seven. In general, the number of plant species (3.9 (mean) ± 1.9 (SD)), animal species (7.5 ± 4.8) and total number of species (11.5 ± 6.1) per network was small (Fig 1a and 1g). Sixty eight percent of networks had a smaller number of species than the average. Most networks had a higher number of insect than plant species, the *A*:*P* ratio varied from one to eightwith a mean of 2.0 ± 1.2. Network size was highly variable, even in matrices with the same number of species. Mean network size was 34.7 ± 33.8 ranging from four to 135 (Fig 1b and 1h). The total number of links was 9.2 ± 6.3 (Fig 1c and 1i). The mean number of links per species was 0.72 ± 0.16 (Fig 1d and 1j). The mean number of links for plant species (2.32 ± 1.27) was significantly higher than for animal species (1.17 ± 0.20) (*t* = 5.88, *P* < 0.001). In general, the total number of links (*I*) in the network and the number of links per species (*L*) increased linearly with the number of species (*S*) (*I*: *R* ^2^ = 0.95, *F*_43_ = 802.5, *P* < 0.001; *L*: *R* ^2^ = 0.45, *F*_43_ = 34.9, *P* < 0.001). Connectance values for most networks were high (0.35 ± 0.12) (Fig 1e and 1k). Nestedness (NODF) values were low, 5.69 ± 7.72, with 52 percent of matrices showing zero values (Fig 1f and lL). The connectance decreased hyperbolically with increasing *M* (*R* ^2^ = 0.68, *F*_43_ = 43.57, *P*< 0.001). NODF increased linearly with *I* (*R* ^2^ = 0.27, *F*_43_ = 16.0, *P*< 0.001).

The observed values of weighted connectance, weighted NODF, interaction strength asymmetry and Shannon’s diversity of interactions differed significantly (*P* < 0.002) from the mean values estimated from random networks in all cases (S2 Table).

### Effects of altitude and time on network metrics

The evaluation of the contribution of altitude and time to the overall variation in network metrics revealed that altitude did not account for a significant fraction of variation in any network property. Time, in turn, accounted for a significant fraction of variation in the number of plant species, and connectance (Table 1). Linear relationships were not detected between network properties and altitude or precipitation. Plant, animal and total number of species, network size, and number of interactions showed quadratic relationships (concave parabola) with precipitation (*P*: *R* ^2^ = 0.16, *F*_43_ = 4.00, *P* = 0.0259; *A*: *R* ^2^ = 0.15, *F*_43_ = 3.54, *P* = 0.0380; *S*: *R* ^2^ = 0.18, *F*_43_ = 4.43, *P* = 0.0181; *M*: *R* ^2^ = 0.19, *F*_43_ = 4.85, *P* = 0.0121; *I*: *R* ^2^ = 0.13, *F*_43_ = 3.02, *P* = 0.0597). Connectance showed a quadratic relationship (convex parabola) with precipitation (*R* ^2^ = 0.16, *F*_43_ = 3.92, *P* = 0.0276).

**Table 1:** Summary of analysis of variance on network properties using altitude and time as factors. *A* = animal species, *P* = plant species, *S* = total number of species, *M* = matrix size (*A* x *P*), *A*:*P* = ratio animal to plant species, *I* = number of interactions, *L* = mean number of links per species, *L*_*P*_ = mean number of links for plant species, *L*_*A*_ = mean number of links for animal species, *C* = connectance, NODF = nestedness. Network properties labeled with an asterisk showed normality and homoscedasticity and were tested with an ANOVA (*F* value is reported). The other variables were analized using a Kruskal-Wallis test (*χ* ^*2*^value is reported).

Effect on	Altitude		Time	
	*F* or *χ* ^*2*^	*P*	*F* or *χ* ^*2*^	*P*
*A*	9.11	0.2448	10.05	0.1853
*P*	1.42*	0.2322	3.29*	0.0105
*S*	9.08	0.2469	2.10*	0.0745
*M*	8.18	0.3164	12.08	0.0977
*A*:*P*	5.23	0.6319	11.62	0.1135
*I*	8.26	0.3099	8.69	0.2755
*L*	1.38*	0.2403	0.83*	0.5732
*L*_*A*_	6.20	0.5162	3.06	0.8789
*L*_*P*_	6.94	0.4341	8.07	0.3257
*C*	0.88*	0.5337	2.43*	0.0432
NODF	7.06	0.4220	4.94	0.6663

### Low vs high altitude variation on network metrics

**Fig 2:**
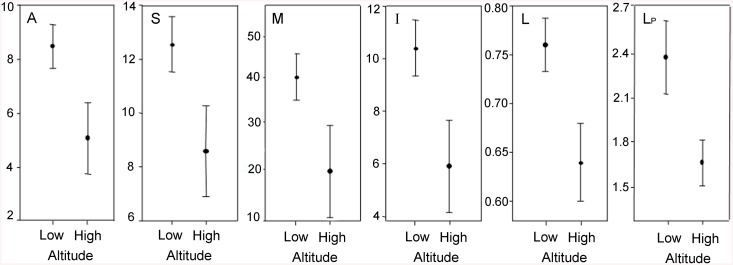
Effects of altitude on properties of plant—flower-visitor networks in a cloud forest in Antioquia, Colombia. Data are presented as mean ± standard errors for network properties at low and high altitude. *A* (Welch *t*—test = 3.05, *P* = 0.004), *S* (Welch *t*—test = 2.61, *P* = 0.013), *M* (Welch *t*—test = 2.52, *P* = 0.016), *I* (Welch *t* test = -.2.99, *P* = 0.005), *L* (*t*—test = -2.31, *P* = 0.026), *L*_*P*_ (*t*—test = 2.14, *P* = 0.038).

Results indicate that the number of animal species, the total number of species, network size, the number of interactions, the mean number of links per species, and the mean number of links per plant species were significantly higher at low altitude (Fig 2). No altitude effect was observed on the number of plant species, the mean number of links per animal species, connectance and nestedness (*P*: *t* = -0.93, *P* = 0.3550, *L*_*A*_: *t* = -1.07, *P* = 0.2898, *C*: *t* = 0.42, *P* = 0.6720, NODF: *t* = 1.64, *P* = 0.1073). Analysis of homogeneity of variances within groups of transects from low and high elevation and their appopiate *t-* test are presented in S3 Table.

### Variation of specialization with altitude

**Fig 3:**
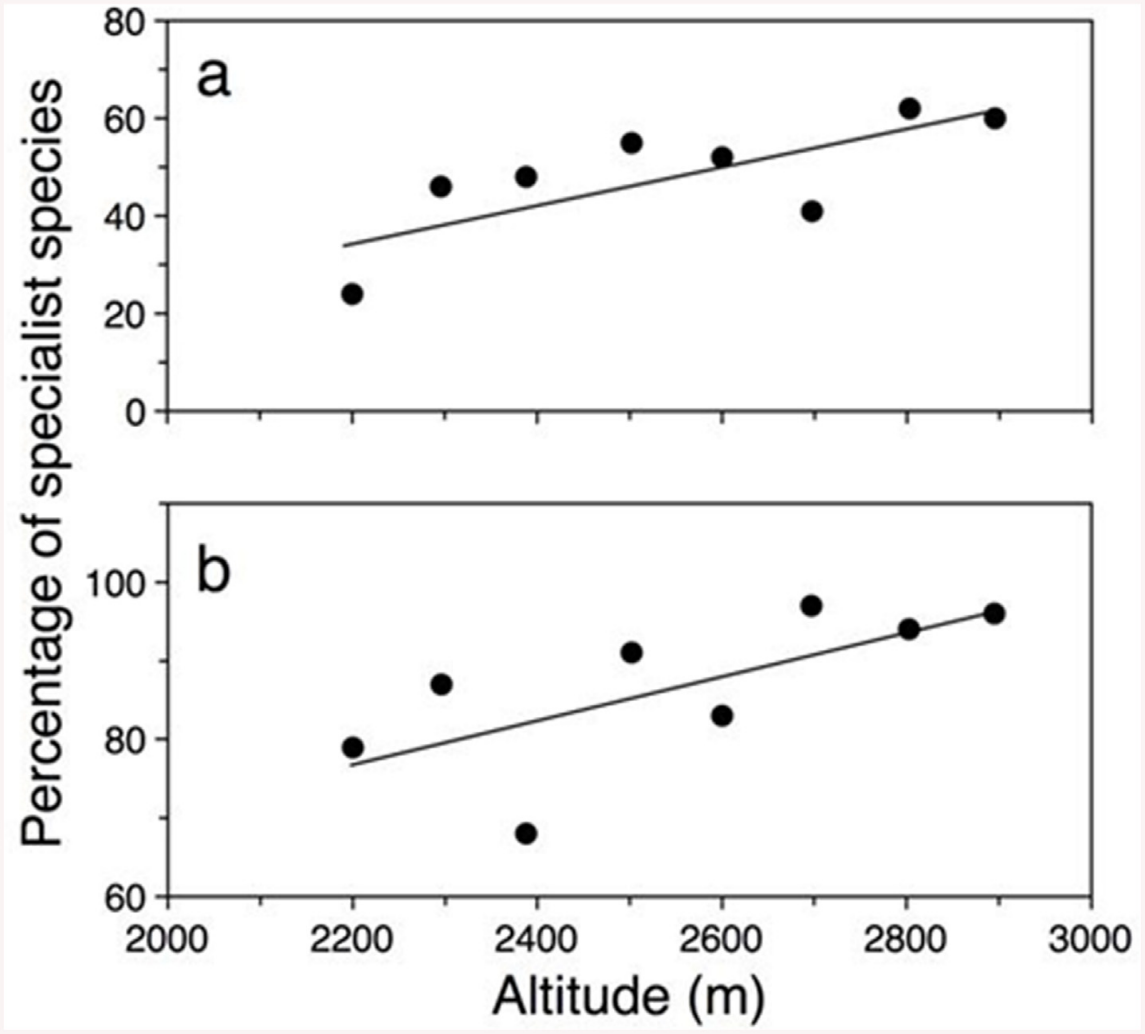
Relationship between altitude and percentage of specialist species. The number and percentage of specialist species was estimated for each altitude. a) Flowering plant species and b) flower-visitors. Equations: % specialist *P* = -0.44 + 0.0003 altitude; % specialist *A* = 0.15 + 0.0002 altitude.

Regarding specialization, the percentage of specialist plant and flower-visitor species increased significantly with altitude for *P* (*R* ^2^ = 0.54, *F*_7_ = 7.22, *P* = 0.036), and marginally significant for *A* (*R* ^2^ = 0.48, *F*_7_ = 5.58, *P* = 0.056) (Fig 3). Although specialization increased at a similar rate in both plants and flower-visitors with altitude, the overall degree of specialization was higher for insects than for plants (*t* = 6.99, *P* < 0.0001). Insects interacting with only one plant species were prevalent, reaching 85 percent of insects per network on average. Plants interacting with only one insect species, in turn, reached 49 percent per network on average. No relationship between degree of specialization and precipitation was detected (*P*: *R* ^2^ = 0.13, *F*_7_ = 0.90, *P* = 0.3782; *A*: *R* ^2^ = 0.06, *F*_7_ = 0.41, *P* = 0.5432).

### Composition and interactions of flowering plant and flower-visitor assemblages

The species composition was highly variable among networks. Both plant and insect species showed high turnover along altitude and time. The mean Bray-Curtis dissimilarity index for insect communities between any pair of networks across space and time was high, reaching 75 percent dissimilarity across each axis. Most insect species were active for only a short period (*ca*. 50% of species were active for less than one month) (S1 Fig). Interestingly, few insect species (*ca*. 2%) were present in all months and all altitudes (e.g., *Phyllotrox* spp., *Frankliniella* spp. and Staphylinidae). Only 4.6% of species were present in six or seven altitudes or months although these were not necessarily contiguous or consecutive, respectively (*Cyclanthura* spp., Nitulidae, Platygastridae, Drosophilidae, Phoridae, and Cecidomidae). Flowering plants had a more restricted distribution than insect species across the altitude gradient and time. Dissimilarity across months (95%) was higher than across altitude (82%). No plant species was present in all altitudes and months. Flowering span was limited for most plant species (*ca*. 55% were registered flowering during one month) (S1 Fig). Only two plant species showed a long-lasting flowering period: *Anthurium yarumalense* was flowering during six months; and *A*. *panduriforme* during five months. Also, only three plant species (2%) were present across four altitudes (*A*.*yarumalense*, *A*.*cupreum*, and *A*. *panduriforme*).

**Fig 4:**
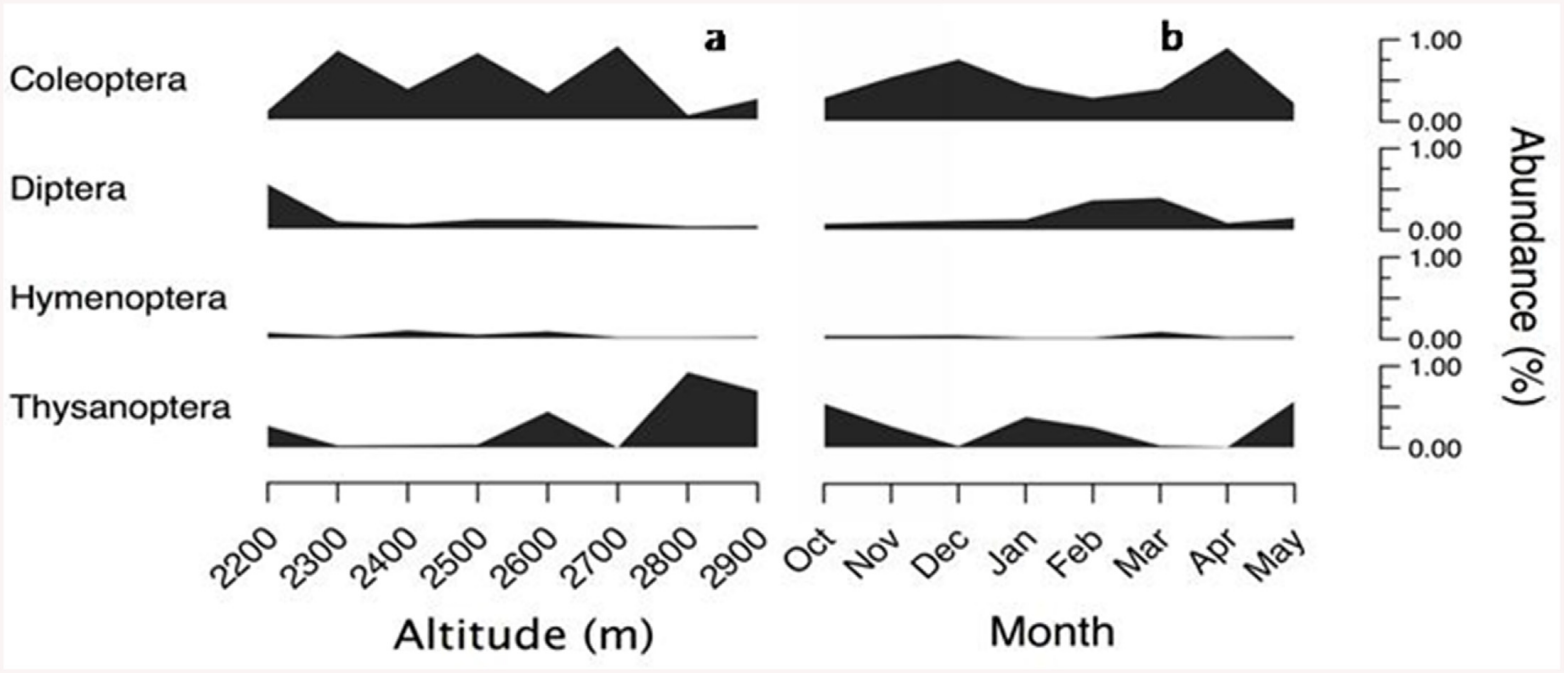
Relative composition of the flower-visitor assemblages to understory flowering plants in a cloud forest in Antioquia, Colombia. a) Networks gathered from eight sampling transects along an altitude gradient between 2200 and 2900 masl pooling time, and b) networks gathered on a monthly basis pooling transects during eight consecutive months.

Flower-visitors belonging to 10 taxonomic orders were recorded during the entire period of study. In general, in spite of some fluctuations, Coleoptera was best represented both in space and time and Hymenoptera and Diptera were poorly represented along the complete gradient (Fig 4a and 4b). A shift in the dominance of taxa along rainy and dry months was observed: Thysanoptera was dominant in the wettest months (October and May), Diptera was dominant in dry months (February and March), and Coleoptera in months with intermediate levels of precipitation (Fig 4a). Only Hymenopteran abundance showed a positive association with precipitation (*R* ^2^ = 0.50, *F*_7_ = 6.11, *P* = 0.048).

The diversity of insects, in terms of the numbers of orders decreased nonlinearly with altitude, but differed between low (T0-T5) and high altitude (T6-T7) (Low: 7.16 ± 0.98, High: 4.5 ± 0.70; *t* = -3.46, *P* = 0.0134). The dominance of insect orders changed across altitude: Coleoptera was significantly more abundant at low than at high altitude (Low: 207.8 ± 62.74, High: 30.5 ± 108.6; *t* = -2.56, *P* = 0.049). On the other hand, the relative abundance of Thysanoptera showed the opposite pattern (Low: 47.5 ± 38.96, High: 233.5 ± 67.48; *t* = 4.69, *P* = 0.003) (Fig 4b).

We recorded 405 interactions in the overall system, most of them found only once through elevation (65%) or through time (68%). Even though the proportion of unique links was high across all altitudes (range: 44–85%), the number of unique interactions decreased with elevation (*R* ^2^ = 0.72, *F*_7_ = 15.54, *P*< 0.008). In contrast, no relationship was observed between the number of unique interactions with precipitation. Interactions present in more than one network were less prevalent. The most frequent interactions were *Anthurium yarumalense*–*Frankliniella* spp. (representing 2.7% of the total number of of interactions), and *Anthurium panduriforme*–*Cyclanthura* spp. (representing 1.2% of the total of interactions).

### Topology of individual vs. cumulative networks

**Fig 5:**
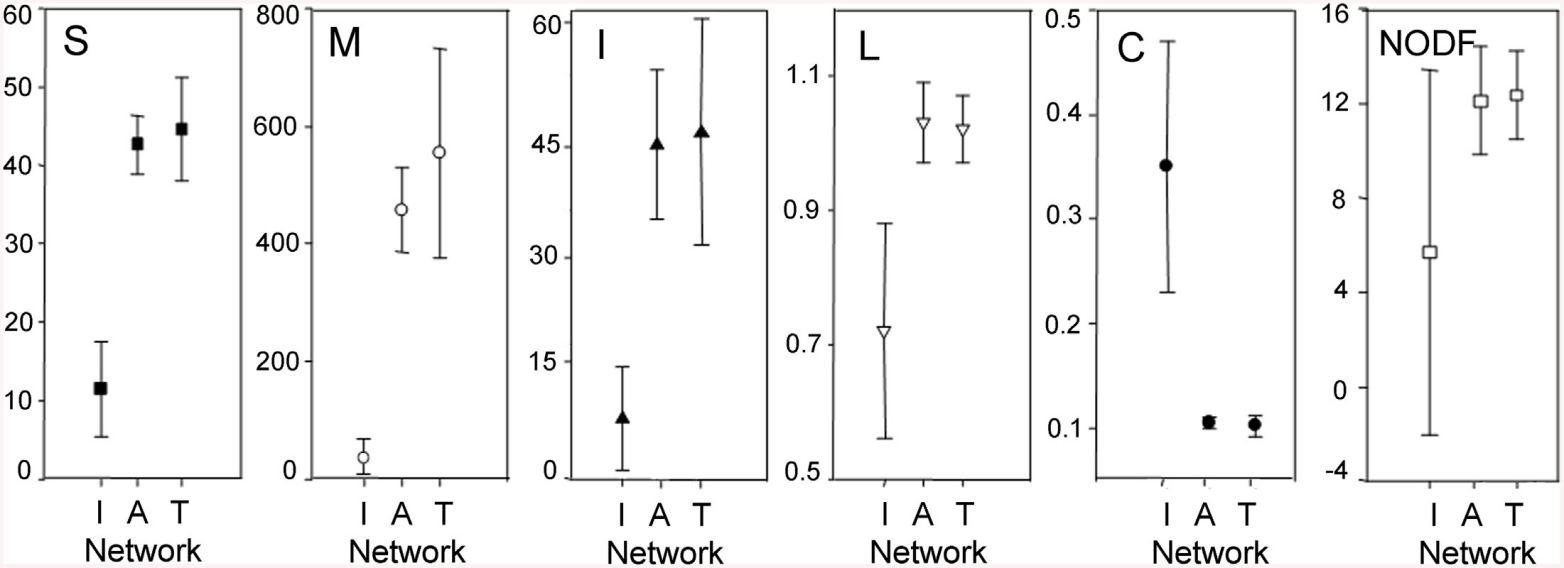
Mean values of topological properties of plant—flower-visitor networks for individual and cumulative networks. Mean value estimated for 44 individual networks (I), eight cumulative “altitude” networks (A), and eight cumulative “time”networks (T). *S* = total number of species, *M* = matrix size, *I* = number of interactions, *L* = mean number of links per species, *C* = connectance, NODF = nestedness. Data are presented as mean ± standard error for each network property.

Pooling all data in one single matrix provided low connectance and nestedness values (0.057 and 6.82, respectively). The mean values for number of species, matrix size, number of interactions, mean number of links per species and nestedness were smaller for individual networks compared to mean values of cumulative networks for each altitude or month (*S*: *χ* ^*2*^ = 33.18, *P* < 0.0001; *M*: *χ* ^*2*^ = 34.07, *P* < 0.0001; *I*: *χ* ^*2*^ = 25.12, *P* < 0.0001; *L*: *χ* ^*2*^ = 25.12, *P* < 0.0001; NODF: *χ* ^*2*^ = 11.07, *P* = 0.0039). In contrast, connectance showed the inverse trend (*χ* ^*2*^ = 34.84, *P* < 0.0001) (Fig 5). Interestingly, the mean values for the majority of variables were largely similar between cumulative spatial (altitude) and temporal (monthly) networks, suggesting that metrics from small network units deviate from similar mean values regardless of the source of variation (S2 Fig). For example, an average cumulative network at any altitude or month consisted of 18 plant and 25 insect species (*S* = 43).

## Discussion

### Network structure

Our analysis of plant—flower-visitor networks along eight altitudinal levels over a period of eight months revealed ample variation in network topology, in the identity of interacting species, and in the composition of plant and flower-visitor assemblages. These results are in agreement with recent studies indicating high turnover of species and interactions among and within seasons and along environmental gradients [8,9,11,45]. In spite of variation, however, some clear patterns emerged for this this mountain forest. First, individual networks are small and the proportion of realized links relative to all potential interactions is high. A characteristic network size in this system consists of four plant and eight insect species. These small networks lie in the low side of network size distribution for other interaction systems in the world. Only 10% of the reviewed networks are of comparable size to the mean size of our networks [1,5]. Second, most networks exhibited a non-nested pattern of organization. The high interconnected pattern of interactions was not related with high nestedness. Nestedness has been described to occur consistently in large networks (*S* > 50) [5]. Although our matrices were smaller than 50 cells, the metric used for nestedness estimation (NODF) is largely insensitive to matrix size, even for those with 25–100 cells [33]. At the same time, nestedness is a robust meausure of network structure which is only slightly influenced by insufficient sampling [46,47]. As matrices in our system have a mean size of 35 cells, our estimates provide reliable nestedness values. Moreover, NODF consistently rejects a nested pattern for different types of random matrices, which is congruent with the mixed random-checkerboard distribution of links in the analyzed matrices [33,48].

However, it is interesting to note that networks become relatively more structured (i.e., nested) as their complexity (number of links) increases, which is consistent with the observed trend in pollination networks showing wide variation in size [5]. The observed pattern of network structure differed from those produced by random networks indicating that the system-specific patterns here described are not produced by random processes.

### Effects of altitude and time on network structure

Variation in network topology was explained in a different way by altitude and time. In the analyzed webs, the number of plant species, network size, number of interactions, and connectance were best explained by time and precipitation. The last three variables have shown to be highly correlated in several pollination networks around the world [1] and also in our system (Pearson *r*_*M-I*_ = 0.93, *r*_*M-C*_ = -0.74, *r*_*C-I*_ = -0.63), and in consequence, it is not surprising they change in a similar way along time. The effect of time is likely associated to the expansion and contraction of networks, probably reflecting the influence of plant phenology on the overall system: an increase in the number of flowering plant species during the transition from rainy to dry season, results in large-sized networks with more interactions, decreasing *C* values. Conversely, during the other months, network size tends to be smaller with a consequent increase in connectance values. These findings may have important consequences for the functioning of this and other systems. For instance, similar pulses in network size and connectance have been previously described during summer and spring months in a temperate forest [14]. Unexpectedly, the altitude gradient did not influence network metrics as described in other studies [1,17,19]. However, network structure and composition changed conspicuously at 2700 m. Networks from altitudes higher than 2700 m were smaller, due to a reduction in the number of insect species. As a consequence, the number of interactions declined at the network and species level above such altitude. It is likely that the well-defined altitudinal divide observed at 2700 masl in this study, associates with changes in the composition and structure of tree vegetation and microclimate described for mountain forests of the Andes [25]. The decline in flower-visitor diversity at high altitude is consistent with findings in other high mountain ecosystems [49,50,51]. Flowering was more prevalent during rainy months, which translated into a higher variation in the number of flowering plants through time than across elevation. Flower-visitor activity and abundance, in turn, were influenced by both altitude and precipitation, indicating that insect dynamics depends strongly on small-scale abiotic conditions [6].

### Composition, interactions and specialization in plant—flower-visitor assemblages

The whole system consists of a high proportion of unique combinations of interacting species with limited periods of activity and a small number of redundant interactions across months or altitudes. Similarly, analyses of small network units revealed that most species within networks interacted with only one species. Insects had a maximum of four interactions. Only three species, *Cyclanthura* spp., *Frankliniella* spp. and Nitulidae, visited four flowering plant species within one month. Plants, in turn, were visited by a larger number of insect-visitor species, i.e., assymetrical networks. One plant species, *Sphaeradenia* spp., was visited by more than 15 insect species during one month, followed by *Anthurium panduriforme* and *Xanthosoma undipes* that were visited by nine and seven insect species, respectively. A high proportion of insect and plant species were replaced every month and at every 100 m elevation. In consequence, networks were very different not only in the composition of species but in detailed aspects related to the identity of species involved in interactions, suggesting that turnover of interactions (*β*-diversity of interactions) is mainly driven by the replacement of species in space and time [52]. Distance decay patterns (i.e., the increased species dissimilarity with geographical distance) have been observed in mutualistic networks [52]. In contrast, a high and consistent turnover of species and interactions over the whole system occurred in our study system without reference to changes in elevation or precipitation [44]. This pattern may result from the presence of a tiny group of permanent species (i.e., core species) and a high proportion of sporadic species. Only few species were present in all elevations and months. This result contrasts with the large core groups observed in other mutualistic pollination systems [8,10,53]. But what factors underly the enormous variation in the composition of species in our system? It is likely that variation in the proportion of different taxa across altitude and months accounts in part for such variability. In our system, Coleoptera and Diptera were involved in most interactions. The dominance of Coleoptera shifted along the altitudinal gradient and with time; in general, there was a replacement of Coleoptera by Thysanoptera at increasing altitude and during rainy months. This result contrasts with other mountain ecosystems in temperate regions where Diptera and Hymenoptera are the most important orders (e.g., [17,49,51,54]) and their shift is often associated with a humidity gradient, with Diptera being dominant at more humid sites and Hymenoptera at dryer sites. Since our study site has a mean annual precipitation of 2000 mm and a relative humidity near 90%, it is likely that abiotic conditions restrict the abundance and diversity of Hymenoptera in this tropical mountain forest [55].

In general, insect species seem to interact in a somewhat erratic pattern with flowering plants. Most insects are minute, often flying short distances and probably taking advantage of local and immediate floral availability in their surroundings. This behavior can keep insects attached with the network even when plant species change across altitude or time [56]. Unlike nested networks, where specialists interact with generalists and the generalist core interacts with generalist species [5], specialized interactions dominated the pattern in this forest mountain system when examined at small scale. This pattern of labile plant-animal interactions may represent an alternative mechanism of interaction in species-poor and highly dynamic networks (i.e., short periods of activity or flowering for the majority of insects and plants). It is likely that species ensure growth and survival under the commonest environmental conditions (i.e., available floral resources), regardless of how many species converge functionally (e.g., exploiting same floral resource) as suggested by Hubbell [57]. However, this does not imply that networks are randomly structured. For instance, in our system, the duration of plant flowering and insect activity followed an exponential and logarithmic frequency distribution, respectively, but contrary to expectations based on random interactions among coexisting species [16], nestedness values were low, suggesting that a random model of interactions was not appropriate to represent our system.

In our study system, plant and animal species seem to interact freely, replacing each other according to enviromental conditions, without any apparent species-specific constraint [58]. Even though we do not have information on the effectiveness of flower-visitors at present, our results suggest that a functional equivalence between very different insects may drive the pollination network dynamics in this system (see also [9]).

### Topology of individual vs. cumulative networks

In this system, cumulative networks were medium-sized, showed low connectance, and lack of nestedness, revealing a relatively unconventional pattern of interaction [1,5]. However, when the whole system was divided into smaller spatial and temporal units, networks showed higher and variable connectances, similar to studies that partitioned networks along time [13,14]. Monthly cumulative networks (i.e., time networks) showed higher variation for number of species, matrix size and number of interactions than spatial cumulative networks (i.e., altitude networks), which is consistent with the trends just described for individual matrices. Also, as expected, connectance decreased in cumulative matrices as they are bigger in size. This reduction has been also observed in plant—pollinator networks from temperate and artic regions.

In general, patterns of interaction from pollination networks from around the world exhibit low connectance values in temperate (mean = 9.6, n = 20), artic (mean = 11.5, n = 5), and tropical grassland/shrub vegetation and low elevation forest communities (mean = 14, n = 4) [1], compared to the estimated connectance values for the analyzed networks in this study. In contrast, estimated nestedness for most networks is higher than those observed in our system and showed similar values in temperate (mean = 0.86, n = 13), artic (mean = 0.86, n = 5), and tropical grassland/shrub vegetation and low elevation forest networks (mean = 0.83, n = 6) [5]. Regarding biotic specialization it has been reported that specialization is lower at tropical than temperate latitudes and that it decreases with increasing local and regional plant diversity: specialization is a response of pollinators to low plant diversity [59]. In our system, plant richness was higher at low elevation [44] and the percentage of specialist insect species visiting flowers was low. The opposite trend was observed at high altitude suggesting that the relationship between plant diversity and specialization may be consistent for mutualistic networks along environmental gradients (i.e, latitude and altitude). However, network properties as connectance and nestedness may exhibit wide variation. In consequence, the organization of network cohesiveness around a central core of interactions may be a contingent property of specific ecosystems rather than an invariant property of pollinator networks.

## Conclusions

Our results contribute to understanding the way plant—flower-visitor network structure and function are influenced by elevation and seasonal precipitation in tropical systems. Our sampling design allowed us to detect variation in the majority of metrics that were analyzed. Two general patterns emerged: (1) Although plant and flower-visitors are active throughout the year, plant—flower-visitor systems tend to be larger and more complex (i.e., have more links) at lower altitudes and high precipitation months; and (2) the specialized but erratic pattern of network organization suggest that high connectance coupled with dynamic and opportunistic interactions may represent an alternative pattern of interaction that confers robustness to perturbations [60,61]. Increasing evidence suggests that network structure may arise from multiple factors, including interaction neutrality, trait matching, spatio-temporal distribution of species, and sampling effects [48,52,62]. Our study adds to this body of evidence through the observation that highly dynamic networks occur in mountain ecosystems where turnover of interactions within individual networks is mainly driven by the replacement of flowering plant species in space and time, which are highly influenced by precipitation. The extent to which opportunistic and erratic species attachment provides structure and influences the persistence of plant—pollinator systems needs to be addressed in future studies. Especially useful will be studies that estimate extinction probabilities of plant and pollinator species in networks with high vs. low partner fidelity.

## Supporting Information

S1 Fig Observed frequency distribution ofduration of activity during a period of eight months in a cloud forest in Antioquia, Colombia. a) For insects visiting flowers (mean = 2.41 months) and b) for flowering plants (mean = 1.88 months).
(TIF)

S2 Fig Altitudinal and temporal variation in topological properties from cumulative networks at eight elevation levels and eight months in a cloud forest in Colombia. Each cumulative “altitude” network was obtained by pooling the eight monthly networks at each specific elevation. Similarly, each cumulative “time” network was obtained by pooling the eight elevation networks at each specific month. (A, G) Total number of species (*S*); (B, H) System size (*M*); (C, I) Total number of links (*I*); (D, J) Mean number of links per species (L); (E, K) Connectance (*C*); (F, L) Nestedness (Metric NODF). Horizontal lines inside boxes indicate the mean value for the cumulative network.
(TIF)

S1 Table Topological properties of 44 plant—flower-visitor networks along an elevation gradient (2200–2900 masl) and during a period of eight months in a cloud forest in Antioquia, Colombia. (DOCX)

S2 Table Summary of *t*—test contrasts between observed and mean null model values for four network indices: weighted connectance, weighted NODF, Interaction strength asymmetry and Shannon’s diversity of interactions. Comparisons were performed for 44 plant—flower-visitor pollinator networksalong an elevation gradient (2200–2900 masl) and during a period of eight months in a cloud forest in Antioquia, Colombia.
(DOCX)

S3 Table Summary of the analysis of homogeneity of variances (Levene´s test) for each network property from groups of networks obtained from low vs. high elevation. (DOCX)
