# Supplementary figures and images for: Topology of Plant - Flower-Visitor Networks in a Tropical Mountain Forest: Insights on the Role of Altitudinal and Temporal Variation
Source: PLoS One. 2015 Oct 29;10(10):e0141804. doi: 10.1371/journal.pone.0141804 (PMC4626383; doi:10.1371/journal.pone.0141804)

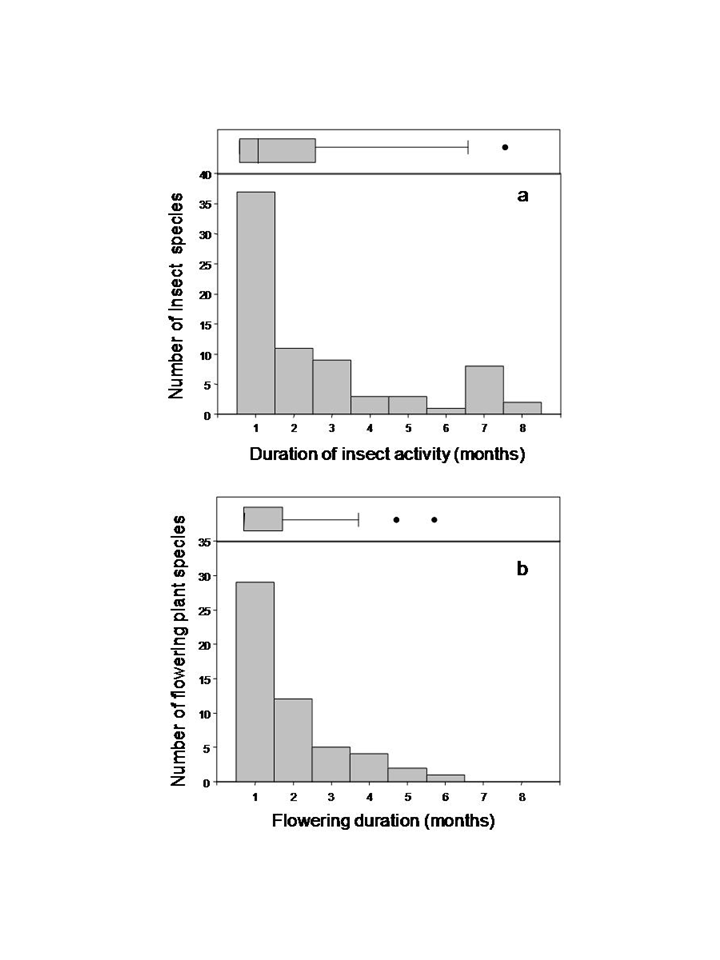

Supplement: S1 Fig — a) For insects visiting flowers (mean = 2.41 months) and b) for flowering plants (mean = 1.88 months). (TIF) [file pone.0141804.s001.tif]

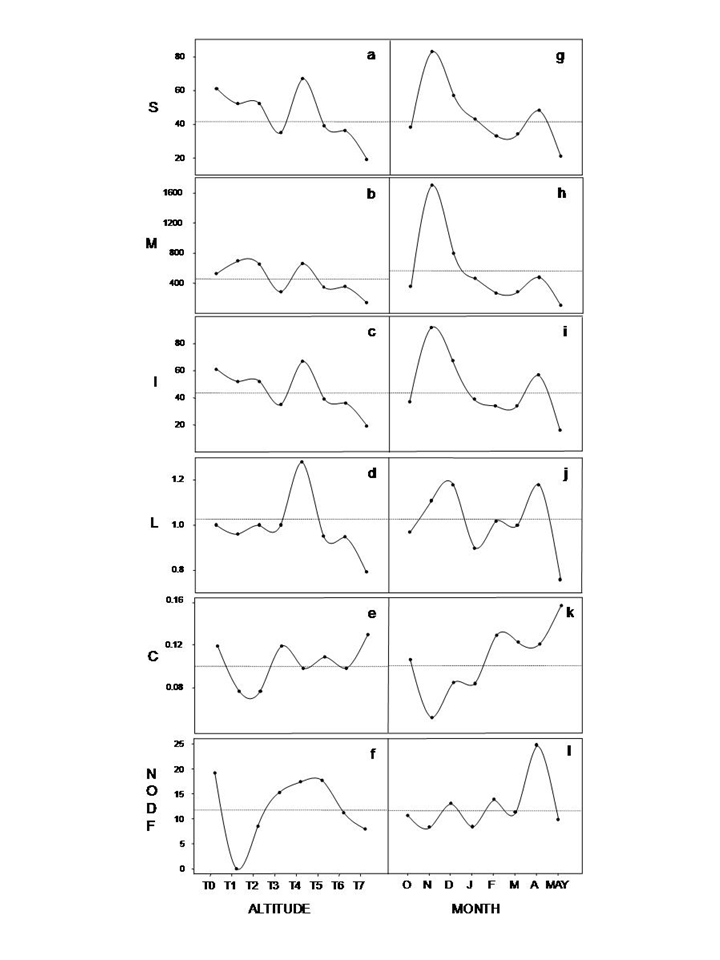

Supplement: S2 Fig — Each cumulative “altitude” network was obtained by pooling the eight monthly networks at each specific elevation. Similarly, each cumulative “time” network was obtained by pooling the eight elevation networks at each specific month. (A, G) Total number of species (S); (B, H) System size (M); (C, I) Total number of links (I); (D, J) Mean number of links per species (L); (E, K) Connectance (C); (F, L) Nestedness (Metric NODF). Horizontal lines inside boxes indicate the mean value for the cumulative network. (TIF) [file pone.0141804.s002.tif]
