# Supplementary material for: Topology of Plant - Flower-Visitor Networks in a Tropical Mountain Forest: Insights on the Role of Altitudinal and Temporal Variation
Source: PLoS One. 2015 Oct 29;10(10):e0141804. doi: 10.1371/journal.pone.0141804 (PMC4626383; doi:10.1371/journal.pone.0141804)
Supplement: S1 Table — (DOCX) [file pone.0141804.s003.docx]

**S1 Table. Topological properties of 44 plant - flower-visitor networks along an elevation gradient (2200 – 2900 masl) and during a period of eight months in a cloud forest in Antioquia, Colombia.**

| **Altitude** | **Month** | ***P*** | ***A*** | ***M*** | ***A:P*** | ***I*** | ***L*** | ***L_P_*** | ***L_A_*** | ***C*** | ***NODF*** |
| --- | --- | --- | --- | --- | --- | --- | --- | --- | --- | --- | --- |
| T0 | N | 9 | 15 | 135 | 1.67 | 24 | 0.95 | 2.67 | 1.60 | 0.18 | 11.70 |
| T0 | D | 5 | 9 | 45 | 1.80 | 13 | 0.92 | 2.60 | 1.44 | 0.29 | 2.17 |
| T0 | E | 5 | 6 | 30 | 1.20 | 8 | 0.72 | 1.60 | 1.33 | 0.27 | 12 |
| T0 | F | 2 | 5 | 10 | 2.50 | 5 | 0.71 | 2.50 | 1.00 | 0.50 | 0 |
| T0 | A | 2 | 7 | 14 | 3.50 | 8 | 0.88 | 4.00 | 1.14 | 0.57 | 0 |
| T0 | May | 3 | 6 | 18 | 2.00 | 7 | 0.77 | 2.33 | 1.17 | 0.39 | 0 |
| T1 | O | 2 | 3 | 6 | 1.50 | 3 | 0.20 | 1.50 | 1.00 | 0.50 | 0 |
| T1 | N | 5 | 20 | 100 | 4.00 | 20 | 0.80 | 4.00 | 1.00 | 0.20 | 0 |
| T1 | D | 6 | 18 | 108 | 3.00 | 21 | 0.87 | 3.50 | 1.17 | 0.19 | 12.60 |
| T1 | E | 3 | 3 | 9 | 1.00 | 4 | 0.66 | 1.33 | 1.33 | 0.44 | 16.67 |
| T1 | F | 5 | 7 | 35 | 1.40 | 9 | 0.75 | 1.80 | 1.29 | 0.26 | 17.74 |
| T2 | O | 3 | 5 | 15 | 1.67 | 6 | 0.75 | 2.00 | 1.20 | 0.40 | 0 |
| T2 | N | 6 | 13 | 78 | 2.17 | 18 | 0.89 | 3.00 | 1.38 | 0.23 | 13.35 |
| T2 | E | 2 | 8 | 16 | 4.00 | 8 | 0.80 | 4.00 | 1.00 | 0.50 | 0 |
| T2 | F | 4 | 5 | 20 | 1.25 | 8 | 0.88 | 2.00 | 1.60 | 0.40 | 18.75 |
| T2 | M | 2 | 4 | 8 | 2.00 | 4 | 0.66 | 2.00 | 1.00 | 0.50 | 0 |
| T2 | A | 4 | 8 | 32 | 2.00 | 9 | 0.75 | 2.25 | 1.13 | 0.28 | 5.88 |
| T2 | May | 2 | 2 | 4 | 1.00 | 2 | 0.50 | 1.00 | 1.00 | 0.50 | 0 |
| T3 | O | 3 | 7 | 21 | 2.33 | 7 | 0.70 | 2.33 | 1.00 | 0.33 | 0 |
| T3 | N | 7 | 10 | 70 | 1.43 | 12 | 0.70 | 1.71 | 1.20 | 0.17 | 6.06 |
| T3 | D | 3 | 5 | 15 | 1.67 | 5 | 0.62 | 1.67 | 1.00 | 0.33 | 0 |
| T3 | A | 2 | 7 | 14 | 3.50 | 8 | 0.88 | 4.00 | 1.14 | 0.57 | 13.64 |
| T4 | O | 4 | 9 | 36 | 2.25 | 13 | 0.92 | 3.25 | 1.44 | 0.36 | 9.52 |
| T4 | N | 6 | 8 | 48 | 1.33 | 9 | 0.64 | 1.50 | 1.13 | 0.19 | 6.98 |
| T4 | D | 9 | 14 | 126 | 1.56 | 24 | 1.04 | 2.67 | 1.71 | 0.19 | 9.93 |
| T4 | E | 4 | 11 | 44 | 2.75 | 16 | 1.00 | 4.00 | 1.45 | 0.36 | 13.66 |
| T4 | F | 3 | 4 | 12 | 1.33 | 4 | 0.57 | 1.33 | 1.00 | 0.33 | 0 |
| T4 | M | 7 | 13 | 91 | 1.86 | 18 | 0.90 | 2.57 | 1.38 | 0.20 | 2.02 |
| T4 | A | 2 | 4 | 8 | 2.00 | 4 | 0.66 | 2.00 | 1.00 | 0.50 | 0 |
| T4 | May | 4 | 5 | 20 | 1.25 | 5 | 0.71 | 1.25 | 1.00 | 0.25 | 0 |
| T5 | D | 4 | 6 | 24 | 1.50 | 6 | 0.60 | 1.50 | 1.00 | 0.25 | 11.43 |
| T5 | A | 3 | 24 | 72 | 8.00 | 25 | 0.92 | 8.33 | 1.04 | 0.35 | 35.15 |
| T6 | O | 4 | 6 | 24 | 1.50 | 9 | 0.80 | 2.25 | 1.50 | 0.38 | 14.29 |
| T6 | N | 6 | 8 | 48 | 1.33 | 11 | 0.78 | 1.83 | 1.38 | 0.23 | 4.65 |
| T6 | D | 6 | 6 | 36 | 1.00 | 7 | 0.58 | 1.17 | 1.17 | 0.19 | 0 |
| T6 | E | 2 | 2 | 4 | 1.00 | 2 | 0.50 | 1.00 | 1.00 | 0.50 | 0 |
| T6 | F | 3 | 5 | 15 | 1.67 | 5 | 0.62 | 1.67 | 1.00 | 0.33 | 0 |
| T6 | M | 3 | 6 | 18 | 2.00 | 6 | 0.66 | 2.00 | 1.00 | 0.33 | 0 |
| T6 | A | 5 | 9 | 45 | 1.80 | 11 | 0.71 | 2.20 | 1.22 | 0.24 | 0 |
| T7 | O | 2 | 4 | 8 | 2.00 | 4 | 0.66 | 2.00 | 1.00 | 0.50 | 0 |
| T7 | N | 2 | 2 | 4 | 1.00 | 2 | 0.50 | 1.00 | 1.00 | 0.50 | 0 |
| T7 | D | 4 | 5 | 20 | 1.25 | 6 | 0.66 | 1.50 | 1.20 | 0.30 | 12.50 |
| T7 | E | 3 | 3 | 9 | 1.00 | 3 | 0.50 | 1.00 | 1.00 | 0.33 | 0 |
| T7 | A | 2 | 5 | 10 | 2.50 | 5 | 0.71 | 2.50 | 1.00 | 0.50 | 0 |

Number of plant species (*P*), number of insect species (*A*), total number of interactions between plants and insects (*I*), network size (*M=A*P*), system symmetry (*A:P* ratio), mean number of links per species (*L*), mean number of interactions across plant and animal species respectively (*L_P_*=*I*/*P* and *L_A_*=*I*/*A*), connectance (*C* = *I*/*M*) and nestedness (*NODF*).
