# Supplementary material for: Topology of Plant - Flower-Visitor Networks in a Tropical Mountain Forest: Insights on the Role of Altitudinal and Temporal Variation
Source: PLoS One. 2015 Oct 29;10(10):e0141804. doi: 10.1371/journal.pone.0141804 (PMC4626383; doi:10.1371/journal.pone.0141804)
Supplement: S2 Table — Comparisons were performed for 44 plant—flower-visitor pollinator networksalong an elevation gradient (2200–2900 masl) and during a period of eight months in a cloud forest in Antioquia, Colombia. (DOCX) [file pone.0141804.s004.docx]

**S2 Table.** Summary of *t -* test contrasts between Observed and mean Null Model values for four network indices: weighted connectance, weighted NODF, interaction strength asymmetry and Shannon’s diversity of interactions. Comparisons were performed for 44 plant - flower-visitor networks along an elevation gradient (2200 – 2900 masl) and during a period of eight months in a cloud forest in Antioquia, Colombia.

| **Network** | **Estimated**  **Value** | **Weighted connectance** | **Weighted NODF** | **Interaction strength asymmetry** | **Shannon’s diversity of interactions** |
| --- | --- | --- | --- | --- | --- |
| T0N | Obs | 0.08890 | 3.63248 | 0.10333 | 0.68607 |
|  | Null mean | 0.18937 | 22.72057 | 0.02555 | 0.20667 |
|  | Lower CI | 0.18851 | 22.44686 | 0.02416 | 0.20409 |
|  | Upper CI | 0.19023 | 22.99427 | 0.02694 | 0.20924 |
|  | *t* | 229.09724 | 136.85141 | -109.80202 | -365.39513 |
|  | *P* | 0.00000 | 0.00000 | 0.00000 | 0.00000 |
| T0D | Obs | 0.12365 | 0.00000 | 0.15833 | 0.78599 |
|  | Null mean | 0.23821 | 18.19384 | 0.04759 | 0.20260 |
|  | Lower CI | 0.23674 | 17.85509 | 0.04467 | 0.19782 |
|  | Upper CI | 0.23969 | 18.53259 | 0.05051 | 0.20738 |
|  | *t* | 152.32720 | 105.39599 | -74.44930 | -239.36814 |
|  | *P* | 0.00000 | 0.00000 | 0.00000 | 0.00000 |
| T0E | Obs | 0.12240 | 8.00000 | -0.10000 | 0.87622 |
|  | Null mean | 0.24304 | 40.90567 | -0.15746 | 0.17841 |
|  | Lower CI | 0.24155 | 40.23725 | -0.16403 | 0.17254 |
|  | Upper CI | 0.24452 | 41.57408 | -0.15090 | 0.18428 |
|  | *t* | 159.62741 | 96.60450 | -17.17563 | -233.25149 |
|  | *P* | 0.00000 | 0.00000 | 0.00004 | 0.00000 |
| T0F | Obs | 0.19620 | 0.00000 | 0.52386 | 1.00000 |
|  | Null mean | 0.37266 | 36.64621 | 0.20469 | 0.06076 |
|  | Lower CI | 0.37200 | 36.18976 | 0.20262 | 0.05775 |
|  | Upper CI | 0.37333 | 37.10267 | 0.20676 | 0.06378 |
|  | *t* | 521.46750 | 157.54580 | -302.73090 | -611.78830 |
|  | *P* | 0.00000 | 0.00000 | 0.00000 | 0.00000 |
| T0A | Obs | 0.20268 | 0.00000 | 0.49278 | 1.00000 |
|  | Null mean | 0.26495 | 47.85864 | 0.34237 | 0.08184 |
|  | Lower CI | 0.26460 | 47.29237 | 0.33781 | 0.07765 |
|  | Upper CI | 0.26529 | 48.42490 | 0.34694 | 0.08603 |
|  | *t* | 353.85597 | 165.85050 | -64.62054 | -429.68016 |
|  | *P* | 0.00000 | 0.00000 | 0.00000 | 0.00000 |
| T0May | Obs | 0.17101 | 11.11111 | 0.01957 | 1.00000 |
|  | Null mean | 0.26074 | 49.54120 | -0.06231 | 0.09001 |
|  | Lower CI | 0.26035 | 48.82296 | -0.06409 | 0.08656 |
|  | Upper CI | 0.26113 | 50.25945 | -0.06053 | 0.09346 |
|  | *t* | 454.88291 | 104.99598 | -90.12355 | -517.61620 |
|  | *P* | 0.00000 | 0.00000 | 0.00000 | 0.00000 |
| T1O | Obs | 0.28392 | 0.00000 | 0.36667 | 1.00000 |
|  | Null mean | 0.39803 | 46.93750 | 0.26974 | 0.05604 |
|  | Lower CI | 0.39671 | 45.31683 | 0.26565 | 0.04816 |
|  | Upper CI | 0.39935 | 48.55817 | 0.27384 | 0.06393 |
|  | *t* | 169.40288 | 56.83304 | -46.41711 | -234.92081 |
|  | *P* | 0.00000 | 0.00000 | 0.00000 | 0.00000 |
| T1N | Obs | 0.16376 | 0.00000 | 0.79642 | 1.00000 |
|  | Null mean | 0.23550 | 36.43824 | 0.46980 | 0.21705 |
|  | Lower CI | 0.23493 | 36.06091 | 0.46689 | 0.21227 |
|  | Upper CI | 0.23608 | 36.81000 | 0.47272 | 0.22182 |
|  | *t* | 244.94670 | 189.50102 | -219.65252 | -321.72783 |
|  | *P* | 0.00000 | 0.00000 | 0.00000 | 0.00000 |
| T1D | Obs | 0.10696 | 8.03571 | 0.53194 | 0.79444 |
|  | Null mean | 0.15891 | 45.08710 | 0.29340 | 0.09499 |
|  | Lower CI | 0.15867 | 44.77762 | 0.29016 | 0.09277 |
|  | Upper CI | 0.15914 | 45.39659 | 0.29665 | 0.09720 |
|  | *t* | 437.04846 | 234.92996 | -144.25355 | -619.47973 |
|  | *P* | 0.00000 | 0.00000 | 0.00000 | 0.00000 |
| T1E | Obs | 0.24399 | 33.33333 | 0.53571 | 0.65349 |
|  | Null mean | 0.28637 | 61.83333 | 0.53816 | 0.10046 |
|  | Lower CI | 0.28514 | 61.06880 | 0.53811 | 0.08497 |
|  | Upper CI | 0.28761 | 62.59787 | 0.53822 | 0.11596 |
|  | *t* | 67.30368 | 73.15134 | 90.83502 | -70.02254 |
|  | *P* | 0.00000 | 0.00000 | 0.00000 | 0.00000 |
| T1F | Obs | 0.13454 | 3.22581 | 0.19660 | 0.86765 |
|  | Null mean | 0.26938 | 39.36855 | 0.07127 | 0.20005 |
|  | Lower CI | 0.26808 | 38.71140 | 0.06828 | 0.19557 |
|  | Upper CI | 0.27069 | 40.02569 | 0.07426 | 0.20454 |
|  | *t* | 202.88326 | 107.92830 | -82.27581 | -292.04779 |
|  | *P* | 0.00000 | 0.00000 | 0.00000 | 0.00000 |
| T2O | Obs | 0.21359 | 19.23077 | 0.01250 | 0.64475 |
|  | Null mean | 0.30701 | 24.95769 | 0.01290 | 0.22433 |
|  | Lower CI | 0.30496 | 24.26402 | 0.00798 | 0.21622 |
|  | Upper CI | 0.30905 | 25.65136 | 0.01782 | 0.23244 |
|  | *t* | 89.72348 | 16.20107 | 0.15948 | -101.71488 |
|  | *P* | 0.00000 | 0.00000 | 0.00000 | 0.00000 |
| T2N | Obs | 0.13791 | 4.56989 | 0.21852 | 0.74921 |
|  | Null mean | 0.20016 | 15.76978 | 0.10800 | 0.31505 |
|  | Lower CI | 0.19896 | 15.48291 | 0.10448 | 0.30991 |
|  | Upper CI | 0.20135 | 16.05666 | 0.11153 | 0.32018 |
|  | *t* | 102.35358 | 76.61129 | -61.46864 | -165.96844 |
|  | *P* | 0.00000 | 0.00000 | 0.00000 | 0.00000 |
| T2E | Obs | 0.17824 | 0.00000 | 0.35000 | 1.00000 |
|  | Null mean | 0.25778 | 36.81448 | 0.19810 | 0.07233 |
|  | Lower CI | 0.25678 | 36.23816 | 0.19487 | 0.06540 |
|  | Upper CI | 0.25879 | 37.39080 | 0.20133 | 0.07926 |
|  | *t* | 155.10626 | 125.35200 | -92.18828 | -262.79442 |
|  | *P* | 0.00000 | 0.00000 | 0.00000 | 0.00000 |
| T2F | Obs | 0.15620 | 6.25000 | 0.03751 | 0.82649 |
|  | Null mean | 0.32417 | 61.59427 | 0.01669 | 0.05655 |
|  | Lower CI | 0.32371 | 60.91265 | 0.01443 | 0.05499 |
|  | Upper CI | 0.32463 | 62.27589 | 0.01896 | 0.05811 |
|  | *t* | 713.32713 | 159.33250 | -18.05422 | -967.67967 |
|  | *P* | 0.00000 | 0.00000 | 0.00000 | 0.00000 |
| T2M | Obs | 0.18795 | 0.00000 | 0.00000 | 1.00000 |
|  | Null mean | 0.22521 | 53.02857 | 0.04254 | 0.06205 |
|  | Lower CI | 0.22484 | 52.44087 | 0.03388 | 0.05276 |
|  | Upper CI | 0.22559 | 53.61627 | 0.05119 | 0.07134 |
|  | *t* | 193.08810 | 177.06420 | 9.64662 | -198.11516 |
|  | *P* | 0.00000 | 0.00000 | 0.04089 | 0.00000 |
| T2A | Obs | 0.15959 | 8.82353 | 0.36111 | 0.84665 |
|  | Null mean | 0.25537 | 38.83657 | 0.20521 | 0.22615 |
|  | Lower CI | 0.25376 | 38.12421 | 0.20212 | 0.21776 |
|  | Upper CI | 0.25699 | 39.54894 | 0.20829 | 0.23453 |
|  | *t* | 116.43411 | 82.67681 | -99.05900 | -145.20423 |
|  | *P* | 0.00000 | 0.00000 | 0.00000 | 0.00000 |
| T3O | Obs | 0.17917 | 0.00000 | 0.59817 | 1.00000 |
|  | Null mean | 0.27456 | 62.36438 | 0.26474 | 0.05564 |
|  | Lower CI | 0.27437 | 61.81866 | 0.26216 | 0.05404 |
|  | Upper CI | 0.27476 | 62.91009 | 0.26731 | 0.05725 |
|  | *t* | 978.72109 | 224.25618 | -254.08589 | -1153.72062 |
|  | *P* | 0.00000 | 0.00000 | 0.00000 | 0.00000 |
| T3N | Obs | 0.11616 | 0.00000 | 0.16667 | 0.55789 |
|  | Null mean | 0.13586 | 1.22273 | 0.11432 | 0.18498 |
|  | Lower CI | 0.13502 | 1.10081 | 0.10427 | 0.17556 |
|  | Upper CI | 0.13670 | 1.34465 | 0.12437 | 0.19440 |
|  | *t* | 46.23245 | 19.68040 | -10.22349 | -77.72206 |
|  | *P* | 0.00000 | 0.00000 | 0.00000 | 0.00000 |
| T3D | Obs | 0.15624 | 0.00000 | 0.41667 | 1.00000 |
|  | Null mean | 0.27539 | 44.93333 | 0.11313 | 0.09442 |
|  | Lower CI | 0.27408 | 43.95085 | 0.10650 | 0.08815 |
|  | Upper CI | 0.27669 | 45.91582 | 0.11976 | 0.10070 |
|  | *t* | 179.38648 | 89.74632 | -89.87585 | -283.16096 |
|  | *P* | 0.00000 | 0.00000 | 0.00000 | 0.00000 |
| T3A | Obs | 0.26084 | 13.63636 | 0.35098 | 0.57799 |
|  | Null mean | 0.27854 | 34.00000 | 0.47784 | 0.12026 |
|  | Lower CI | 0.27791 | 33.41851 | 0.47117 | 0.10776 |
|  | Upper CI | 0.27916 | 34.58149 | 0.48452 | 0.13276 |
|  | *t* | 55.26952 | 68.72128 | 37.29169 | -71.87034 |
|  | *P* | 0.00000 | 0.00000 | 0.00000 | 0.00000 |
| T4O | Obs | 0.13980 | 5.95238 | 0.22400 | 0.64168 |
|  | Null mean | 0.18699 | 42.43758 | 0.00574 | 0.12280 |
|  | Lower CI | 0.18662 | 42.06199 | 0.00208 | 0.11967 |
|  | Upper CI | 0.18737 | 42.81317 | 0.00940 | 0.12593 |
|  | *t* | 247.26155 | 190.62205 | -117.04882 | -325.38849 |
|  | *P* | 0.00000 | 0.00000 | 0.00000 | 0.00000 |
| T4N | Obs | 0.10090 | 4.65116 | 0.32440 | 0.95151 |
|  | Null mean | 0.26640 | 40.79845 | 0.04868 | 0.16414 |
|  | Lower CI | 0.26538 | 40.21107 | 0.04685 | 0.16102 |
|  | Upper CI | 0.26743 | 41.38582 | 0.05051 | 0.16726 |
|  | *t* | 317.04975 | 120.76332 | -295.56229 | -494.78862 |
|  | *P* | 0.00000 | 0.00000 | 0.00000 | 0.00000 |
| T4D | Obs | 0.13381 | 15.20468 | 0.16039 | 0.59925 |
|  | Null mean | 0.22999 | 42.16082 | 0.05732 | 0.14938 |
|  | Lower CI | 0.22937 | 41.77267 | 0.05599 | 0.14758 |
|  | Upper CI | 0.23062 | 42.54898 | 0.05866 | 0.15118 |
|  | *t* | 302.99568 | 136.27807 | -151.28469 | -490.66777 |
|  | *P* | 0.00000 | 0.00000 | 0.00000 | 0.00000 |
| T4E | Obs | 0.14796 | 14.70588 | 0.07354 | 0.63781 |
|  | Null mean | 0.24496 | 33.14990 | -0.00728 | 0.14319 |
|  | Lower CI | 0.24418 | 32.79709 | -0.00928 | 0.13990 |
|  | Upper CI | 0.24574 | 33.50272 | -0.00528 | 0.14648 |
|  | *t* | 244.11984 | 102.58515 | -79.26455 | -295.10803 |
|  | *P* | 0.00000 | 0.00000 | 0.00000 | 0.00000 |
| T4F | Obs | 0.16404 | 0.00000 | 0.00000 | 1.00000 |
|  | Null mean | 0.31108 | 7.70556 | 0.00927 | 0.20505 |
|  | Lower CI | 0.30853 | 7.19057 | 0.00493 | 0.19386 |
|  | Upper CI | 0.31362 | 8.22054 | 0.01362 | 0.21623 |
|  | *t* | 113.31510 | 29.36173 | 4.18538 | -139.46303 |
|  | *P* | 0.00000 | 0.00000 | 0.00000 | 0.00000 |
| T4M | Obs | 0.07627 | 1.01010 | 0.03717 | 0.85188 |
|  | Null mean | 0.15031 | 29.73656 | -0.05520 | 0.13248 |
|  | Lower CI | 0.14961 | 29.38995 | -0.05853 | 0.12866 |
|  | Upper CI | 0.15102 | 30.08316 | -0.05188 | 0.13631 |
|  | *t* | 204.99744 | 162.63788 | -54.46069 | -369.07362 |
|  | *P* | 0.00000 | 0.00000 | 0.00000 | 0.00000 |
| T4A | Obs | 0.22024 | 0.00000 | 0.40000 | 1.00000 |
|  | Null mean | 0.38461 | 30.70476 | 0.12421 | 0.15514 |
|  | Lower CI | 0.38286 | 30.02533 | 0.11875 | 0.14693 |
|  | Upper CI | 0.38636 | 31.38419 | 0.12967 | 0.16336 |
|  | *t* | 184.61342 | 88.68242 | -99.08444 | -201.81177 |
|  | *P* | 0.00000 | 0.00000 | 0.00000 | 0.00000 |
| T4May | Obs | 0.26546 | 0.00000 | 0.53333 | 1.00000 |
|  | Null mean | 0.33635 | 39.49545 | 0.42870 | 0.09531 |
|  | Lower CI | 0.33511 | 38.41765 | 0.42130 | 0.08369 |
|  | Upper CI | 0.33758 | 40.57326 | 0.43609 | 0.10692 |
|  | *t* | 112.62095 | 71.90851 | -27.76237 | -152.84269 |
|  | *P* | 0.00000 | 0.00000 | 0.00000 | 0.00000 |
| T5D | Obs | 0.13501 | 0.00000 | 0.50725 | 1.00000 |
|  | Null mean | 0.16210 | 43.55769 | 0.02451 | 0.05750 |
|  | Lower CI | 0.16187 | 43.22738 | 0.01419 | 0.05013 |
|  | Upper CI | 0.16232 | 43.88800 | 0.03483 | 0.06488 |
|  | *t* | 236.15870 | 258.77416 | -91.78039 | -250.77065 |
|  | *P* | 0.00000 | 0.00000 | 0.00000 | 0.00000 |
| T5A | Obs | 0.12292 | 2.56016 | 0.76215 | 0.98030 |
|  | Null mean | 0.14873 | 41.33097 | 0.54420 | 0.04176 |
|  | Lower CI | 0.14867 | 41.04631 | 0.54124 | 0.03974 |
|  | Upper CI | 0.14880 | 41.61564 | 0.54717 | 0.04378 |
|  | *t* | 755.58003 | 267.26684 | -144.23520 | -911.79630 |
|  | *P* | 0.00000 | 0.00000 | 0.00000 | 0.00000 |
| T6O | Obs | 0.15438 | 14.28571 | -0.43056 | 0.89100 |
|  | Null mean | 0.18489 | 40.07222 | -0.11159 | 0.10728 |
|  | Lower CI | 0.18431 | 39.66303 | -0.12096 | 0.09858 |
|  | Upper CI | 0.18546 | 40.48141 | -0.10222 | 0.11599 |
|  | *t* | 104.77357 | 123.66339 | 66.79157 | -176.66341 |
|  | *P* | 0.00000 | 0.00000 | 0.00000 | 0.00000 |
| T6N | Obs | 0.14917 | 6.97674 | -0.60476 | 0.93074 |
|  | Null mean | 0.17370 | 36.69360 | -0.54443 | 0.09329 |
|  | Lower CI | 0.17351 | 36.48701 | -0.54702 | 0.08814 |
|  | Upper CI | 0.17388 | 36.90020 | -0.54184 | 0.09844 |
|  | *t* | 259.59286 | 282.26966 | 45.71509 | -319.16804 |
|  | *P* | 0.00000 | 0.00000 | 0.00000 | 0.00000 |
| T6D | Obs | 0.10703 | 0.00000 | -0.51515 | 1.00000 |
|  | Null mean | 0.16629 | 34.65444 | -0.64573 | 0.13083 |
|  | Lower CI | 0.16550 | 34.17435 | 0.65006 | 0.12236 |
|  | Upper CI | 0.16707 | 35.13454 | -0.64139 | 0.13931 |
|  | *t* | 148.31347 | 141.64567 | -59.13926 | -201.26167 |
|  | *P* | 0.00000 | 0.00000 | 0.00000 | 0.00000 |
| T6E | Obs | 0.10703 | 0.00000 | -0.51515 | 1.00000 |
|  | Null mean | 0.16609 | 34.69056 | -0.64725 | 0.13373 |
|  | Lower CI | 0.16529 | 34.21468 | -0.65154 | 0.12520 |
|  | Upper CI | 0.16688 | 35.16643 | -0.64297 | 0.14225 |
|  | *t* | 146.63353 | 143.05270 | -60.46860 | -199.49146 |
|  | *P* | 0.00000 | 0.00000 | 0.00000 | 0.00000 |
| T6F | Obs | 0.13501 | 0.00000 | 0.50725 | 1.00000 |
|  | Null mean | 0.16223 | 43.74615 | 0.02700 | 0.05320 |
|  | Lower CI | 0.16201 | 43.42541 | 0.01684 | 0.04609 |
|  | Upper CI | 0.16245 | 44.06689 | 0.03716 | 0.06032 |
|  | *t* | 245.39387 | 267.64724 | -92.76674 | -261.06058 |
|  | *P* | 0.00000 | 0.00000 | 0.00000 | 0.00000 |
| T6M | Obs | 0.16445 | 0.00000 | 0.00000 | 1.00000 |
|  | Null mean | 0.26748 | 9.06759 | 0.03595 | 0.25399 |
|  | Lower CI | 0.26555 | 8.72588 | 0.03020 | 0.24281 |
|  | Upper CI | 0.26942 | 9.40931 | 0.04169 | 0.26516 |
|  | *t* | 104.46336 | 52.07163 | 12.27472 | -130.99296 |
|  | *P* | 0.00000 | 0.00000 | 0.00233 | 0.00000 |
| T6A | Obs | NA | 8.69565 | -0.33700 | 0.95211 |
|  | Null mean | NA | 39.15319 | -0.25524 | 0.08639 |
|  | Lower CI | NA | 38.76161 | -0.25634 | 0.08130 |
|  | Upper CI | NA | 39.54477 | -0.25413 | 0.09147 |
|  | *t* | NA | 152.63220 | 145.31226 | -333.89820 |
|  | *P* | NA | 0.00000 | 0.00000 | 0.00000 |
| T7O | Obs | 0.27247 | 0.00000 | 0.37037 | 1.00000 |
|  | Null mean | 0.36695 | 42.06429 | 0.31455 | 0.13051 |
|  | Lower CI | 0.36526 | 40.72646 | 0.30576 | 0.11870 |
|  | Upper CI | 0.36863 | 43.40212 | 0.32334 | 0.14233 |
|  | *t* | 110.22816 | 61.70032 | -12.46603 | -144.39502 |
|  | *P* | 0.00000 | 0.00000 | 0.00295 | 0.00000 |
| T7D | Obs | 0.13482 | 6.25000 | 0.21081 | 0.92998 |
|  | Null mean | 0.28527 | 55.59792 | 0.06812 | 0.10942 |
|  | Lower CI | 0.28440 | 54.91068 | 0.06206 | 0.10592 |
|  | Upper CI | 0.28613 | 56.28515 | 0.07418 | 0.11292 |
|  | *t* | 340.84846 | 140.90817 | -46.19772 | -459.93106 |
|  | *P* | 0.00000 | 0.00000 | 0.00000 | 0.00000 |
| T7E | Obs | 0.16667 | 0.00000 | 0.00000 | 1.00000 |
|  | Null mean | 0.20815 | 63.00000 | 0.00000 | 0.00000 |
|  | Lower CI | 0.20775 | 62.34000 | NA | -0.00096 |
|  | Upper CI | 0.20855 | 63.65000 | NA | 0.00296 |
|  | *t* | 202.30760 | 189.00000 | NA | -999.00000 |
|  | *P* | 0.00000 | 0.00000 | NA | 0.00000 |
| T7A | Obs | 0.16575 | 0.00000 | 0.37037 | 1.00000 |
|  | Null mean | 0.24744 | 53.21364 | 0.17246 | 0.00936 |
|  | Lower CI | 0.24717 | 52.63361 | 0.16951 | 0.00782 |
|  | Upper CI | 0.24771 | 53.79366 | 0.17540 | 0.01089 |
|  | *t* | 591.55990 | 180.03330 | -131.86220 | -1268.02710 |
|  | *P* | 0.00000 | 0.00000 | 0.00000 | 0.00000 |

Obs: observed value. Null mean: mean null model value. Lower CI: lower 95% confidence interval. Upper CI: upper 95% confidence interval. *t*: *t-*statistic. *P*: *P*-value of *t* statistic.
