# Supplementary material for: Topology of Plant - Flower-Visitor Networks in a Tropical Mountain Forest: Insights on the Role of Altitudinal and Temporal Variation
Source: PLoS One. 2015 Oct 29;10(10):e0141804. doi: 10.1371/journal.pone.0141804 (PMC4626383; doi:10.1371/journal.pone.0141804)
Supplement: S3 Table — (DOCX) [file pone.0141804.s005.docx]

**S3 Table.** Summary of the analysis of homogeneity of variances (Levene´s test) for each network property from groups of networks obtained from low vs. high elevation. Network properties labeled with an asterisk were tested using a Welch *t* - test. The other variables were analyzed using a *t -* test. *A* = animal species, *P* = plant species, *S* = total number of species, *M* = matrix size (*A* x *P*), *A*:*P* = ratio animal to plant species, *I* = number of interactions, *L* = mean number of links per species, *L_P_* = mean number of links for plant species, *L_A_* = mean number of links for animal species, *C* = connectance, NODF = nestedness.

| Variable | Levene´s test | |  | *t* - test | |
| --- | --- | --- | --- | --- | --- |
|  | *F* | *P* |  | *t* | *P* |
| *A* | 5.46 | 0.0243 |  | 3.05* | 0.0040 |
| *P* | 0.69 | 0.4100 |  | 0.93 | 0.3550 |
| *S* | 5.01 | 0.0305 |  | 2.61* | 0.0128 |
| *M* | 7.24 | 0.0102 |  | 2.52* | 0.0156 |
| *A:P* | 2.34 | 0.1332 |  | 1.74 | 0.0882 |
| *I* | 8.07 | 0.0069 |  | 2.99* | 0.0047 |
| *L* | 1.77 | 0.1895 |  | 2.31 | 0.0259 |
| *L_A_* | 0.77 | 0.3840 |  | 1.07 | 0.2898 |
| *L_P_* | 2.81 | 0.1006 |  | 2.14 | 0.0379 |
| *C* | 0.52 | 0.4712 |  | 0.42 | 0.6720 |
| NODF | 4.05 | 0.0528 |  | 1.64 | 0.1073 |
